# Zika virus induced microcephaly and aberrant hematopoietic cell differentiation modeled in novel neonatal humanized mice

**DOI:** 10.3389/fimmu.2023.1060959

**Published:** 2023-02-07

**Authors:** Kimberly Schmitt, James Z. Curlin, Leila Remling-Mulder, Tawfik Aboellail, Ramesh Akkina

**Affiliations:** Department of Microbiology, Immunology, and Pathology, Colorado State University, Fort Collins, CO, United States

**Keywords:** humanized mouse model for Zika microcephaly, A dual-purpose hu-mouse model for Zika, Zika virus and hematopoiesis, Zika virus and aberrant B cell development, Neonatal hu-mouse model for Zika, Zika viral effects on human CD34^+^ HSC, Modeling Zika pathologies in newborns, Neuronal impairment by Zika in childhood

## Abstract

**Introduction:**

Immunocompetent and immunocompromised murine models have been instrumental in answering important questions regarding ZIKV pathogenesis and vertical transmission. However, mimicking human congenital zika syndrome (CZS) characteristics in these murine models has been less than optimal and does not address the potential viral effects on the human immune system.

**Methods:**

Here, we utilized neonatal humanized Rag2^-/-^γc^-/-^ mice to model CZS and evaluate the potential viral effects on the differentiation of human hematopoietic stem cells *in vivo*. Newborn Rag2^-/-^γc^-/-^ mice were engrafted with ZIKV-infected hematopoietic stem cells (HSC) and monitored for symptoms and lesions.

**Results:**

Within 13 days, mice displayed outward clinical symptoms that encompassed stunted growth, hunched posture, ruffled fur, and ocular defects. Striking gross pathologies in the brain and visceral organs were noted. Our results also confirmed that ZIKV actively infected human CD34^+^ hematopoietic stem cells and restricted the development of terminally differentiated B cells. Histologically, there was multifocal mineralization in several different regions of the brain together with ZIKV antigen co-localization. Diffuse necrosis of pyramidal neurons was seen with collapse of the hippocampal formation.

**Discussion:**

Overall, this model recapitulated ZIKV microcephaly and CZS together with viral adverse effects on the human immune cell ontogeny thus providing a unique *in vivo* model to assess the efficacy of novel therapeutics and immune interventions.

## 1 Introduction

The adverse impact of ZIKV on pregnant women resulting in children born with microcephaly due to the capacity of ZIKV to cross the placental barrier and cause intrauterine infection has been well documented (1, 2). Head computed tomography and magnetic resonance imaging analysis has revealed extensive damage to the brain structures such as cortical calcifications, hypogyration, ventriculomegaly, and white matter brain abnormalities (3, 4). Neuropathological analysis and autopsies of fetuses and infants from infected mothers during pregnancy have shown numerous calcifications in the fetal brain and severe microcephaly (5, 6). Additionally, infants with congenital Zika infection have presented with a high rate of ocular abnormalities due to retinal lesions, chorioretinal atrophy, and optic nerve abnormalities (6, 7). The constellation of symptoms due to ZIKV infection that include microcephaly, neuromotor deficits, epileptic seizures, tremors, calcifications, and arthrogryposis are now collectively known as congenital Zika syndrome (CZS) (8, 9). Many of these children that have developed CZS from *in utero* exposure will suffer lifelong long-term effects of severe intellectual disability resulting in significant financial burdens (10).

Various mouse models of ZIKV infection have been developed utilizing embryonic, newborn, young, and adult mice. Immunocompetent and immunocompromised mouse models have been very effective in modeling ZIKV pathogenesis and vertical transmission. The most frequently used mouse models that mimic ZIKV infection-mediated microcephaly and CZS during pregnancy thus far have been both wild type mice and interferon (IFN) signaling deficient mice (11). These models confirmed that ZIKV could compromise the integrity of the placenta and that the severity of congenital infection in the fetus correlated with the time of ZIKV infection during pregnancy (12–14).

Mouse models with genetic deficiencies in the IFN-pathway, which include A129, AG129, *Stat2* knockout, *hStat2* knock-in, *Tlr7^-/-^, Mavs^-/-^, Irf3^-/-^/Irf7^-/-^* double knockout, and *Irf3^-/-^/Irf5^-/-^/Irf7^-/-^* triple knockout mice, have been extensively used to study ZIKV pregnancy and tropism as well as define the role type I IFN plays during ZIKV pathogenesis (12, 15–24). Deficiency in the IFN response with mouse models like A129, *Ifnar1^-/-^*, and *Irf3^-/-^Irf5^-/-^Irf7^-/-^* mice resulted in clinical manifestations with detectable viral loads in multiple tissues such as the brain, spinal cord, spleen, ovaries, and testis within 5-10 days post-inoculation (15, 16, 22, 25). Additionally, vertical transmission of ZIKV has been shown in *Ifnar1^-/-^* pregnant mice that are susceptible to ZIKV infection *via* subcutaneous inoculation which mimics a mosquito bite and results in placental virus transfer into developing fetal brains (15). Fetuses (*Ifnar1^-/+^)* from the ZIKV-infected *Ifnar1^-/-^* dams mated with WT sires showed decreased head and body size and an increased reabsorption rate demonstrating a limitation to these studies. Interestingly, both higher viral replication titers in the placenta and fetal head, brain damage and littermate survival were commonly seen in homozygous (*Ifnar1^-/-^*) IFN deficient mice when compared to heterozygous (*Ifnar1^-/+^)* mice subcutaneously or intravaginally infected with ZIKV (12, 15, 24, 26, 27). During ZIKV infection of human pregnant women, more severe fetal abnormalities correlate with the first and second trimester (28, 29). Infection of several IFN deficient mouse models with various ZIKV strains has demonstrated that placental insufficiency and fetal demise occurred during early gestation (embryonic day 6, E6) whereas infection during mid-gestation (E9) produced reduced cranial dimensions, and infection later in pregnancy (E12) resulted in no fetal disease (15, 30–32). However, one of the major drawbacks of these immunocompromised models is the lack of essential immune system components and response during ZIKV infection.

The broad spectrum of neurodevelopmental abnormalities seen in infants and children as well as the pathological effects detected in aborted human fetal brains have been modeled more effectively using immunocompetent wild type mice (11). Previous studies have shown that cerebroventricular space/LV ZIKV inoculation led to replication of ZIKV in mouse fetal brains targeting the neural progenitor cells (NPCs), resulting in massive neuronal cell death followed by cortical thinning and reduced brain size post-birth (33, 34). These mouse studies not only indicate that ZIKV can cross the blood-brain barrier, but also effectively recapitulate most of the pathologies associated with human CZS by the progressive activation of microglia, astrogliosis, neuronal death, and the disruption of glial cell development (33, 34). However, a severe limitation to this model is that death of the newborns occurs shortly after birth. Direct inoculation of ZIKV into the amniotic fluid or uterus has also been shown to result in spontaneous abortions, intracranial calcifications, spinal cord defects and motor neuron and ocular malformations (35, 36).

Immunocompetent neonatal mice post-birth can also be used as key models to study brain developmental processes by mimicking the second and third trimesters of human fetal development (37). Intracranial inoculation of postnatal mice resulted in reduced brain size, cortical calcifications, massive neuronal cell death and activation of microglia and astrocytes (38–40). Subcutaneous or intraperitoneal injection of 7- to 8-day old neonatal mice leads to central nervous system pathology and partial lethality whereas subcutaneous inoculation of 1-day old neonatal mice resulted in nonfatal neurological complications such as tremors, ataxia, and seizures followed by neurodegeneration in the cerebellum accompanied by CD4^+^ and CD8^+^ T cell infiltration into the brain (41–43). Overall, these neonatal mouse models are apt for defining mechanisms of ZIKV pathogenesis as an alternative to immunocompromised adult mice given the critical role that the immune system plays in viral and brain pathogenesis. However, the neurodevelopmental complications, viral pathogenesis, and mortality due to ZIKV infection are all contingent on the strain of mouse used, embryonic or postnatal age at the time of ZIKV inoculation and virus strain making it difficult to evaluate the long-term neurodevelopmental and behavioral complications.

Since only a subset of immunocompetent mice are susceptible to ZIKV infection and able to survive postnatally, and interferon deficient mice lack a critical component of the innate immune system, we developed a Rag2^-/-^γc^-/-^ neonatal humanized mouse model for CZS. Humanized mice, such as hu-HSC mice, are intrahepatically engrafted with human blood forming CD34^+^ cells that produce a broad spectrum of hematopoietic stem cells that encompass T cells, B cells, monocytes/macrophages, and dendritic cells at various stages of development and displays viral susceptibility (44–49). Utilizing this hu-HSC model, we are able to infect CD34^+^ HSC with ZIKV prior to intrahepatic engraftment of 1- to 3-day old Rag2^-/-^γc^-/-^ mice to determine which human immune cells traffic ZIKV to the various organs such as the brain, spleen, and bone marrow, thus providing a unique platform to evaluate ZIKV pathogenesis, immunity and CZS. Our results show that these ZIKV neonatal humanized mice display discernible clinical signs of ZIKV infection and restricted development of CD34^+^ HSC into terminally differentiated B cells thus demonstrating the pathogenic effects of ZIKV on the developing hematopoietic system. Furthermore, we were able to histologically recapitulate CZS thus reinforcing the validity of this model for future therapeutic and vaccine candidate studies.

## 2 Materials and methods

### 2.1 ZIKV propagation

The Puerto Rico strain of ZIKV (PRVABC59) used for this study was a kind gift from Dr. Rushika Perera at Colorado State University. Low passage ZIKV (MOI of 0.01) was propagated by infecting Vero cells in DMEM supplemented with 2% FBS, 1x antibiotic-antimycotic mix (Thermo Fisher Scientific, Waltham, MA) and 2 mM _L_-glutamine. Virus was harvested when the cytopathic effect observed was severe (≥ 50% cell death). The viral supernatant was 0.45 μm filtered and tittered by plaque assay.

### 2.2 Preparation of human hematopoietic CD34^+^ Cells

Human fetal liver-derived CD34^+^ hematopoietic stem cells (HSCs) were column purified and cultured as previously described in IMDM media containing 20% FBS supplemented with 10 ng/mL of IL-3, IL-6 and SCF (R & D Systems, Inc., Minneapolis, MN) for 24 h (50).

### 2.3 Neonatal humanized Rag2^-/-^γc^-/-^ mouse preparation and infection

Freshly isolated CD34^+^ HSC described above were infected with low passage ZIKV PRVABC59 at an MOI of 3 for 4 hours at 37°C in IMDM media containing 2% FBS supplemented with 10 ng/mL of IL-3, IL-6 and SCF (R & D Systems, Inc., Minneapolis, MN). As a negative/mock control, CD34^+^ HSC were incubated with virus-free media containing the cytokines described above only. The ZIKV-infected and uninfected CD34^+^ cells were then incubated with heat inactivated human DENV patient antiserum (1:100), known to cross-neutralize ZIKV PRVABC59, for an hour to remove all free ZIKV in the culture. The cells were washed three times prior to intrahepatic injection into 1-to 3-day old Rag2^-/-^γc^-/-^ neonatal mice (0.5 x 10^6^ ZIKV-infected or uninfected CD34^+^ cells/mouse) preconditioned by irradiation at 350 rads (ZIKV-infected n = 11; uninfected n = 6). Mice were closely monitored for the presence of ZIKV-related symptoms and euthanized as symptoms progressed. Noticeable ZIKV-related symptoms, such as hunched posture, ruffled fur, uncontrollable shaking, hindleg paralysis and weight loss among others occurred within 13-21 days. Mice were maintained at the Colorado State University Painter Animal Center, and all studies conducted in this publication have been reviewed and approved by the CSU Institutional Animal Care and Use Committee.

### 2.4 Measurement of Zika viral load by qRT-PCR

At the time of euthanasia, blood from cardiac puncture was collected and plasma was separated. Viral RNA was extracted using the E.Z.N.A. Viral RNA kit (Omega Bio-tek, Norcross, GA) according to the manufacturer’s instructions. Viral loads were determined by qRT-PCR utilizing the iScript One-Step RT-PCR kit with SYBR green (Bio-Rad, Hercules, CA) with optimized virus-specific primers as previously described (45, 51). All samples were run using a Bio-Rad C1000 Thermal Cycler with a CFX 9 Real-Time System (Bio-Rad, Hercules, CA) and the following cycling conditions: 50°C for 10 min, 95°C for 3 min, followed by 40 cycles of 95°C for 15 s and 58°C for 60 s. The standard curve was prepared using a series of 10-fold dilutions of ZIKV PRVABC59 at a known concentration. The sensitivity of this assay was 1,000 copies per mL (1 copy per μL).

### 2.5 Histopathology

The neonatal hu-mice were euthanized between 14-21 days post-engraftment as described above. The head and brain were examined for gross pathology as well as assessed for weight and caliper measurements prior to fixation in 10% neutral buffered formalin (10% NBF). The remaining abdominal and thoracic cavities were inspected for gross pathology, with each organ (lungs, heart, kidneys, liver, spleen) being weighed and caliper measured prior to fixation in 10% NBF. Fixed specimens were transferred to the Colorado State University Diagnostic Laboratories, BSL-2 for trimming. The oral cavity, salivary glands, olfactory bulb, cerebrum, cerebellum, and brain stem were thoroughly inspected for gross lesions. Trimmed sagittal and coronal sections of the brain and other visceral organs were processed, embedded in paraffin wax to obtain 4–5 μm sections that were stained with hematoxylin and eosin for blinded evaluation by the pathologist using the Nikon 80i Eclipse microscope (Nikon Microscopy, Melville, NY).

### 2.6 Immunohistochemistry

Sections from hemi-brains were stained using ultraView Universal Alkaline Phosphatase Red Detection kit (Ventana Medical Systems, Inc., Tucson, AZ). Heat-induced epitope retrieval was performed on a Leica Biosystems BOND-III IHC Automated Stainer using the Bond Epitope Retrieval Solution (Leica Biosystems Division of Leica Microsystems, Inc., Buffalo Grove, IL) for 20 minutes. Viral envelope antigen was detected using a purified monoclonal mouse 4G2 Pan-Flavivirus Envelope-specific antibody (CDC, Fort Collins). Labeling was performed on an automated staining platform. Fast Red (Fast Red Substrate System, Dako North America Inc., Carpinteria, CA; PAX-5) was used as chromogen and slides were counterstained with hematoxylin. Immunoreactions were visualized by a single pathologist in a blinded fashion. In all cases, normal and reactive mouse brain sections incubated with primary antibodies were used as positive immunohistochemical controls. Negative controls were incubated in diluent consisting of Tris-buffered saline with carrier protein and homologous nonimmune sera. All sequential steps of the immunostaining procedure were performed on negative controls following incubation.

### 2.7 Immunofluorescence staining and tissue imaging

Paraffin embedded tissue sections were stained using ZIKA Polyclonal rabbit anti-ZIKV pre-membrane and envelope MR-766 (PrM-Env) antibody (CDC, Fort Collins, CO) was used at a 1:1,000 dilution using a Leica Bond RX^m^ automated staining instrument (Leica Biosystems Division of Leica Microsystems, Inc., Buffalo Grove, IL) following permeabilization using 0.01% Triton X diluted in Tris-buffered saline (TBS). Fast red (Fast Red Substrate System, Dako North America Inc., Carpinteria, CA; PAX-5) and 3,3′-diaminobenzidine (DAB; Liquid DAB+ Substrate, Dako North America Inc., Carpinteria, CA) were used as chromogens and sections were counterstained with hematoxylin. Immunoreactions were visualized using commercial detection systems for PowerVision™ poly-AP IHC reagents using Biotin-free, anti-Rabbit primary antibodies (BOND Polymer Refine Detection System, Leica Biosystems Division of Leica Microsystems, Inc., Buffalo Grove, IL). In all cases, negative controls were incubated in diluent consisting of Tris-buffered saline with carrier protein and homologous nonimmune sera. All sequential steps of the immunostaining procedure were performed on negative controls following incubation. Images were captured using a Nikon 80i Eclipse microscope (Nikon Instruments, Inc., Melville, NY). Images were collected and regions of interest subjectively quantified as follows: immunoreactive staining in less than 10% of the area of interest (equivocal or minimal, +/-); Immunoreactivity in 10-25% of the area of interest (mild, +); immunoreactivity in 25-50% of the area of interest (moderate, ++); and immunoreactivity in >50% of the area of interest (marked, +++).

### 2.8 Flow cytometric analysis

Prior to fixation with 10% NBF, a small piece of tissue from the spleen as well as bone marrow and PBMC were harvested and processed into cell suspensions by collagenase and/or mechanical dissociation at the time of euthanasia. Red blood cells were then lysed using the Whole Blood Erythrocyte Lysing kit per the manufacturer’s instructions (R & D Systems, Minneapolis, MN). These cells were used in a flow cytometric assay as described below to identify the different types and subsets of cells from the neonatal hu-mice infected with ZIKV. The fluorochrome conjugated antibodies used for the spleen and PBMC were as follows: CD14-APC/H7 (clone MΦP9), CD19-BB515 (clone HIB19), CD20-PE/CF594 (clone 2H7), and CD3-PE (HIT3a) (all antibodies obtained from BD Biosciences, San Jose, CA). The panel for the bone marrow was as follows: CD14-APC/H7 (clone MΦP9), CD19-BB515 (clone HIB19), and CD34-PE (clone 563) (all antibodies obtained from BD Biosciences, San Jose, CA). Briefly, cell suspensions were incubated for 5 min at room temperature with Fc block (human serum, mouse serum, and anti-CD16/32) in a FACS staining buffer (0.5% BSA and 0.05% sodium azide in 1x PBS). Cell surface marker panels listed below were performed in the FACS staining buffer at a 1:50 dilution for 30 min at 4°C then washed. Cells were then either fixed in 1% paraformaldehyde to assess total cell populations in the ZIKV-infected and mock-infected neonatal mice or fixed/permeabilized using the BD Cytofix/Cytoperm Fixation/Permeabilization kit (BD Biosciences, Sane Jose, CA) to additionally stain intracellularly for ZIKV. For intracellular ZIKV staining, cells were then incubated in Fixation/Permeabilization solution for 20 minutes at 4°C. All subsequent antibody steps were performed in the dark with BD Perm/Wash Buffer supplemented with 2% goat serum and Fc block (as described above) per the manufacturer’s instructions. Cells were first incubated with a 1:500 dilution of monoclonal anti-flavivirus group antigen 4G2 antibody (Millipore, Billerica, MA) or a mouse IgG2a isotype control antibody (Millipore, Billerica, MA) at 4°C for 1 h. Cells were and then resuspended with goat anti-mouse IgG2a conjugated A647 secondary antibody for 45 min at 4°C. Cells were washed, fixed in 1% paraformaldehyde, and assessed using the BD FACSCelesta flow cytometer (BD Biosciences, Sane Jose, CA). For all flow cytometry experiments, the data was analyzed on FlowJo v10.0.7 software (FlowJo LLC, Ashland, OR).

### 2.9 Statistical analysis

Data was analyzed and statistical tests were run using GraphPad Prism 8 software (GraphPad Software, San Diego, CA). Student’s *t*-test were preformed where indicated and statistical significance was defined as p ≤ 0.05. All graphs are presented as mean ± standard deviation.

## 3 Results

### 3.1 ZIKV-infected humanized Rag2^-/-^γc^-/-^ neonatal mice develop CZS

**Figure 1:**
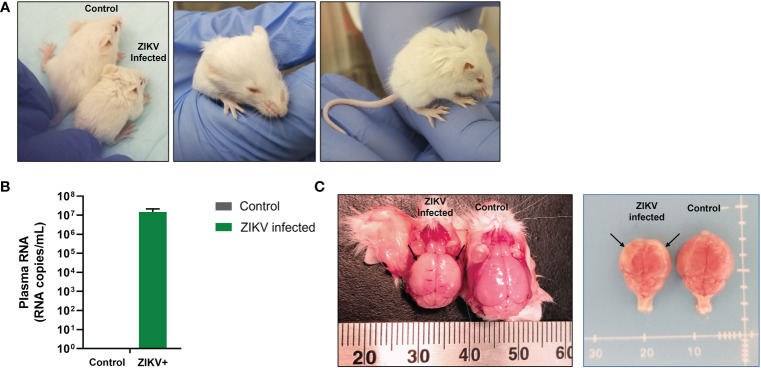
Growth retardation and gross microcephaly in ZIKV-infected neonatal hu-mice. One-to-three-day old neonatal Balb/c-Rag2^-/-^γc^-/-^ mice were irradiated and inoculated intrahepatically with ZIKV-infected or uninfected CD34^+^ HSC. **(A)** ZIKV-infected neonatal hu-mice displayed stunted growth (left), underdeveloped eyes (middle), and hunched posture (right) when compared to age-matched uninfected control hu-mice. **(B)** ZIKV plasma viremia in neonatal mice at time of euthanasia using qRT-PCR. **(C)** Brains isolated from these ZIKV-infected neonatal hu-mice showed significant indicators of microcephaly (left) when compared to age-matched uninfected control hu-mice (right). Significant diminution was observed in the overall size of the cerebral hemispheres, which appear paler (black arrows) due to the thinning of the neocortex, neuronal necrosis, and mineralization. Representative images are shown of day 17 post-engraftment.

Immunocompetent and immunocompromised murine models help decipher important questions such as how ZIKV induces damage to the developing brain and the potential role of the immune system in dampening this damage. To answer these questions, without the need for pregnant dams or viral injection into fetal brains, we developed a humanized Rag2^-/-^γc^-/-^ neonatal mouse model to study the pathogenesis of ZIKV CZS and potential effects of the virus on hematopoietic cells. Human CD34^+^ hematopoietic stem cells (HSC) were infected *in vitro* with ZIKV (Puerto Rican strain PRVABC59) and injected i/p into 1- to 3-day old immunodeficient neonatal Rag2^-/-^γc^-/-^ mice. Mice were closely monitored for ZIKV-related neurological symptoms such as cerebellar ataxia and were euthanized as symptoms progressed. Within 13 days post-engraftment, ZIKV-infected mice displayed clinical symptoms of infection that included stunted growth and/or small stature, hunched posture, ruffled fur, proprioceptive deficits, tremors, and/or hindleg paralysis, microphthalmia, and reduced weight gain compared to age-matched uninfected/mock control mice (**Figure 1A**).

**Figure 2:**
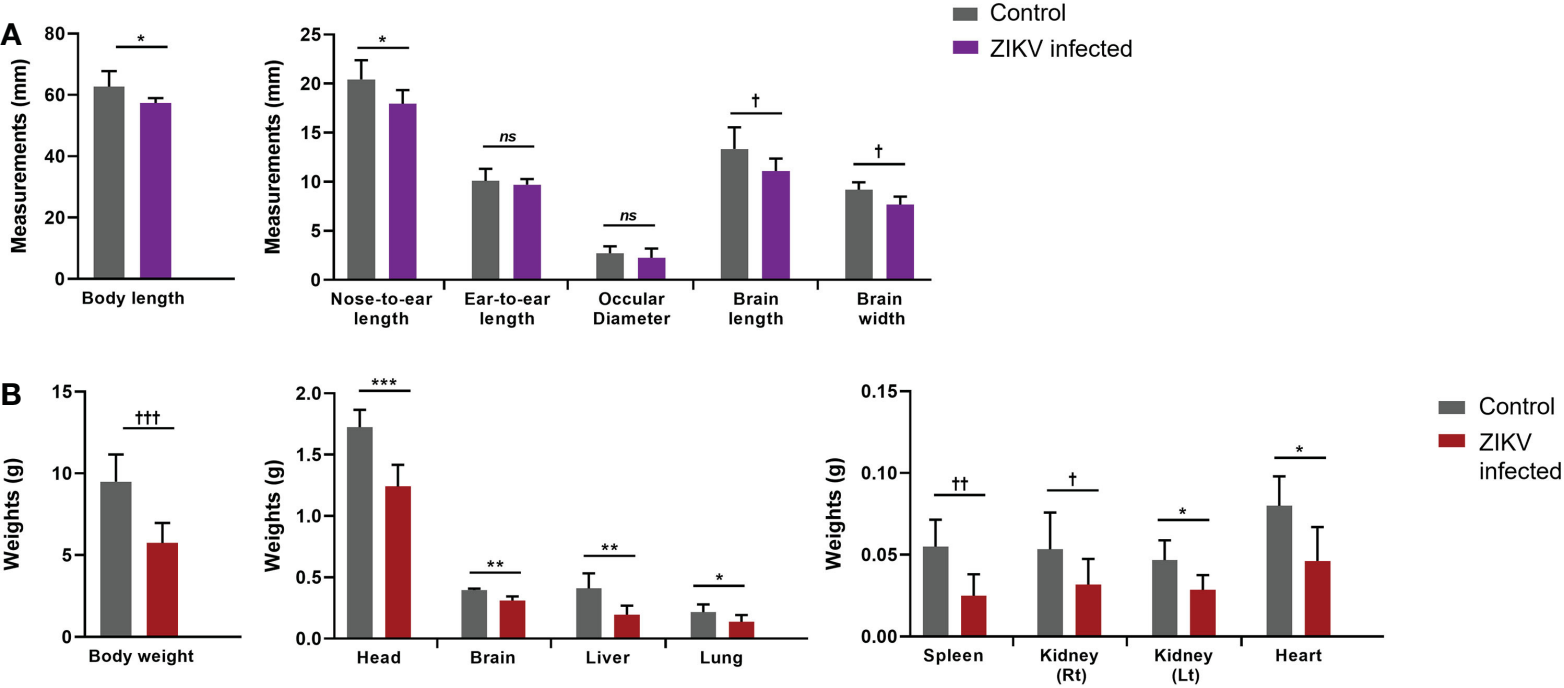
Arrested development of whole body and organs determined by gross measurements of ZIKV-infected neonatal hu-mice. One-to-three-day old neonatal Balb/c-Rag2^-/-^γc^-/-^ mice were irradiated and inoculated intrahepatically with ZIKV-infected CD34^+^ HSC. **(A)** Measurements (mm) of the neonatal ZIKV-infected (n = 11) and uninfected (n = 6) mice were taken upon euthanasia (13-21 days post-inoculation) using a caliper and averaged for comparison. **(B)** The total mass of the neonatal mice as well as their constitutive tissues were weighed (g) and averaged for comparison. The neonatal mice displayed significant gross weight and measurement differences when uninfected and ZIKV-infected neonatal mice were compared (ns, no significance, Two-tailed Student’s *t*-test †p<0.05; *p<0.01; ††p<0.005; **p<0.001; †††p<0.0005; ***p<0.0001).

Mice were euthanized between 13-21 days post-infection as they became moribund with ZIKV. At the time of euthanasia, whole blood was collected to determine plasma viral loads using qRT-PCR. All mice infected with ZIKV showed plasma viral titers greater than 10^6^ RNA copies per mL (average 6.51 x 10^6^ RNA copies per mL; **Figure 1B**). We also found ZIKV RNA in the liver and pituitary gland of several mice (>10^5^ RNA copies/mL) (data not shown). Additionally, gross pathologies were observed, and gross measurements were taken of the whole mouse and individual organs. Gross pathology of the mice showed visible periarticular hemorrhages in the hindlegs and occasionally calvarial hemorrhage upon brain removal from the skull (data not shown). The brains from the ZIKV-infected neonatal mice showed significant diminution in the size of the cerebral hemispheres when compared to age-matched control/mock mice (**Figure 1C**). Pallor of the cerebral hemispheres was observed due to the thinning of the neocortex, neuronal necrosis, and mineralization. Overall, ZIKV-infected neonatal hu-mice were significantly smaller in stature with regards to body and nose-to-ear lengths compared to the age-matched controls (**Figure 2A**). Additionally, the brain length and width were also significantly reduced. The body weight and organs of ZIKV-infected neonatal hu-mice were significantly less than the age-matched controls (**Figure 2B**). This included the head, lungs, liver, spleen, kidneys, and heart. Collectively, these data showed that ZIKV pathogenesis and CZS could be recapitulated in our humanized Rag2^-/-^γc^-/-^ neonatal mouse model.

### 3.2 ZIKV infection has adverse effects on HSC and impairs their differentiation into B cells

ZIKV has been shown to infect human monocytes, macrophages, and dendritic cells (52–56). Previously, we showed that ZIKV is capable of infecting human B cells (CD19^+^), myeloid (CD14^+^), and fetal derived hematopoietic stem cells (HSC, CD34^+^) using humanized mice (45). However, since the potential effects of ZIKV infection on CD34^+^ HSC and their differentiation into terminal end-stage cells *in vivo* were not elucidated, we sought to evaluate this question.

**Figure 3:**
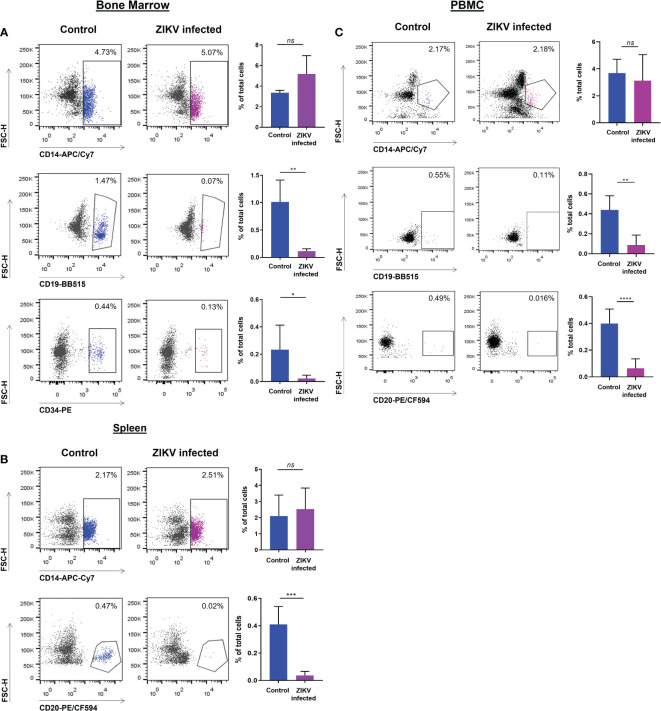
ZIKV infection of CD34^+^ HSC cells restricts development into terminally differentiated cells. PBMC as well as bone marrow and spleen-derived cell suspensions from ZIKV-infected and uninfected humanized neonatal mice at the time of euthanasia were stained with different fluorophore-labeled antibodies and analyzed by flow cytometric analysis. Values are presented as the percentage of total cells assessed. **(A)** Myeloid (CD14^+^), B cells (CD19^+^), and HSC (CD34^+^) cellular markers were gated on cells from the bone marrow. **(B)** Cellular markers for myeloid (CD14^+^) and B cells (CD20^+^) were gated on cells from the spleen. **(C)** Myeloid (CD14^+^) and B cell (CD19^+^, CD20^+^) markers were gated on PBMC. Representative flow plots are shown to the left and the averaged flow data is displayed on the right. Statistical analysis was performed using a Student’s *t*-Test (ns, no significance; *p<0.01; **p<0.005; ***p<0.001; ****p<0.0005).

We determined if ZIKV infection of fetal CD34^+^ HSC affects their development *in vivo* into terminally differentiated immune cells. First, PBMC, and cell suspensions from the spleen and bone marrow were each analyzed by flow cytometry at the time of euthanasia to evaluate the effect of ZIKV infection on different cell populations. The CD19^+^ B cells in the bone marrow (**Figure 3A**) and blood (**Figure 3C**) were significantly reduced in the ZIKV-infected humanized neonatal mice compared to controls.

**Figure 4:**
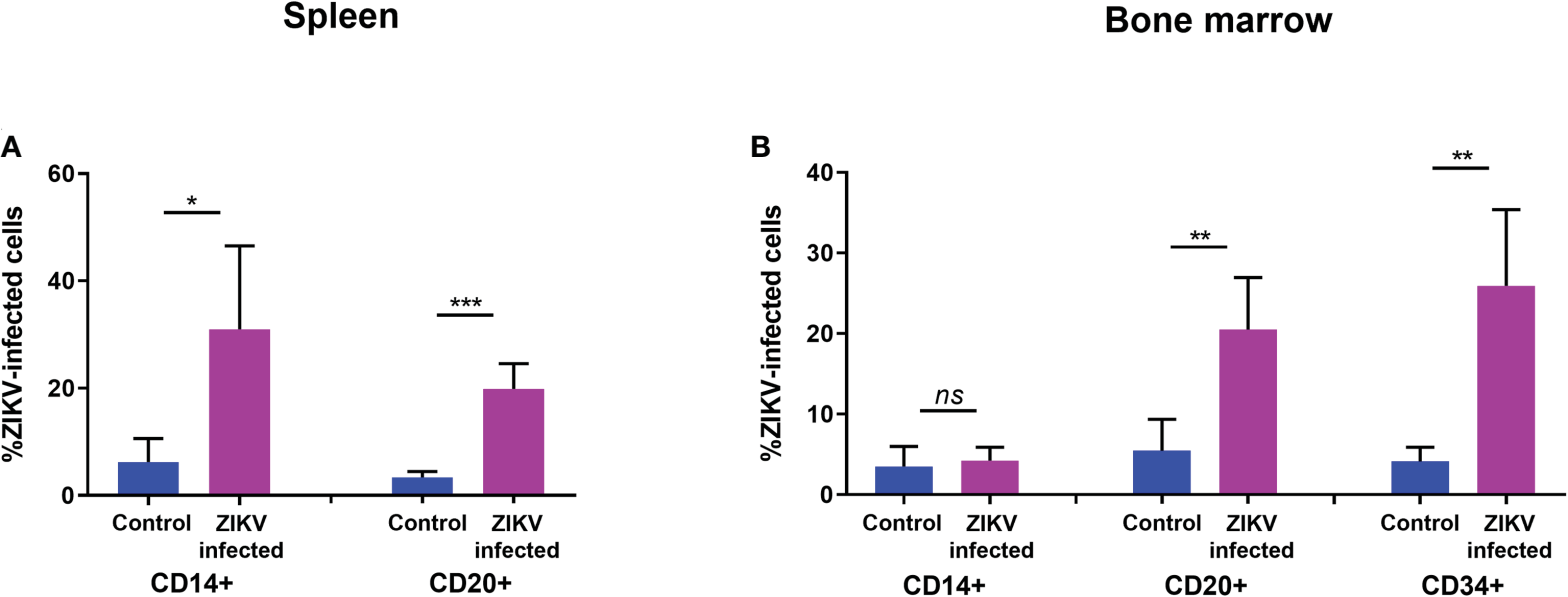
ZIKV infection of specific human immune cells in the spleen and bone marrow of neonatal mice. **(A)** spleen and **(B)** bone marrow cell suspensions from ZIKV-infected and uninfected humanized neonatal mice at the time of euthanasia were stained with different fluorophore-labeled antibodies using flow cytometric analysis. Samples were pre-gated on indicated cellular markers for myeloid (CD14^+^), B cells (CD19^+^ and CD20^+^), or HSC (CD34^+^) and intracellular staining for ZIKV (4G2+) was examined within each gate. The averaged flow data is shown. Statistical analysis was performed using a Student’s *t*-Test (ns, no significance; *p<0.01; **p<0.001; ***p<0.0001).

Similarly, the levels of CD20^+^ B cells in the spleen (**Figure 3B**) and in PBMC (**Figure 3C**) were also significantly reduced. However, no significant reduction was seen in the levels of CD14^+^ monocytes in the bone marrow, spleen, or blood (**Figure 3**). The percentage of CD34^+^ HSC was significantly decreased in ZIKV-infected neonatal humanized mice when compared to age-matched controls indicating the effects of the virus on these cells. We stained for ZIKV intracellularly and confirmed that human CD14^+^ and CD20^+^ cells of the spleen (**Figure 4A**) and bone marrow (**Figure 4B**) are prone to productive ZIKV infection in these neonatal humanized mice. Additionally, CD34^+^ cells were also found to be infected with ZIKV, which has implications for the role of ZIKV on immune cell ontogeny and differentiation (**Figure 4B**).

### 3.3 ZIKV infection in humanized Rag2^-/-^γc^-/-^ neonatal mice leads to brain pathologies

**Figure 5:**
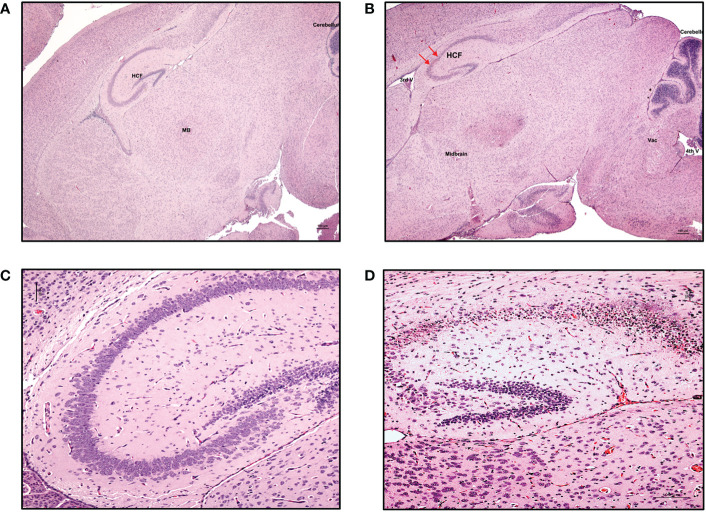
Neuronal necrosis in the hippocampus was consistently observed in ZIKV-infected neonatal hu-mice. Hematoxylin-eosin staining of the hippocampus of age-matched uninfected control and ZIKV-infected neonatal hu-mice. **(A)** The neonatal age-matched uninfected control hu-mouse showing a normal cortex, hippocampus formation (HCF), striatum, and midbrain (MB). **(B)** ZIKV-infected neonatal hu-mouse at 15-days post-engraftment showing hippocampal neuronal necrosis (red arrows). **(C)** The neonatal age-matched uninfected control hu-mouse showing normal pyramidal cell neurons with large, open-faced nuclei and a normal hippocampal molecular layer. **(D)** ZIKV-infected neonatal hu-mouse at 15 days post-engraftment showing diffuse necrosis of the pyramidal neurons and subsequent collapse of the *cornu Ammonis* CA1, CA2 and CA3 layers of the HCF. Images **(A)** and **(B)** were taken at magnification 100x, while **(C)** and **(D)** were taken at magnification 200x. The black scale bar in each image represents 100 µm. Representative images are shown. The red arrows refer to the hippocampal neuronal necrosis.

**Figure 6:**
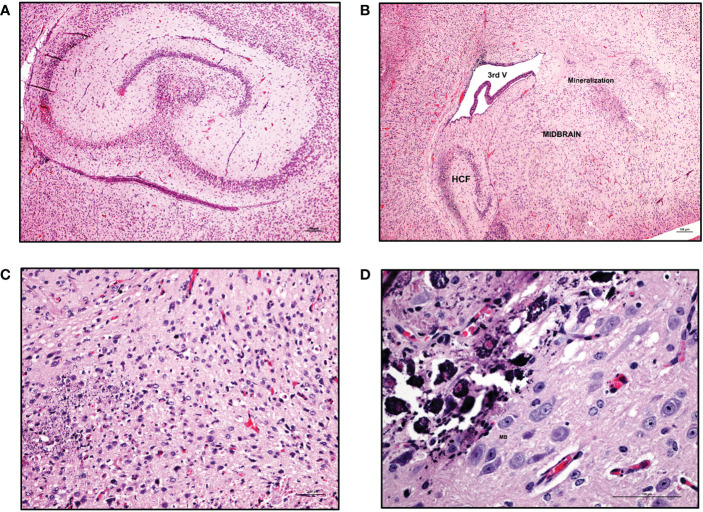
Mineralization in various regions of CNS in ZIKV-infected neonatal hu-mice. Hematoxylin-eosin staining of hemi-brains in ZIKV-infected neonatal hu-mice. **(A)** Bilateral necrosis of the hippocampus, focal hemorrhage and mineralization in ZIKV-infected neonatal hu-mice. **(B)** Multifocal mineralization in the cerebral cortex, thalamus, striatum, and hippocampal formation (HCF). **(C)** Magnified view of widespread neuronal necrosis and mineralization leading to focal disruption of cytoarchitecture of striatum basal nuclei. **(D)** Marked and more advanced mineral encrustation of the neurons in the midbrain substantia nigra was observed. Images **(A)** and **(B)** were taken at a magnification of 100x, while the magnification of images **(C)** and **(D)** were taken at a magnification of 200x and 400x respectively. The black scale bar in each image represents 100 µm. Representative images are shown.

**Figure 7:**
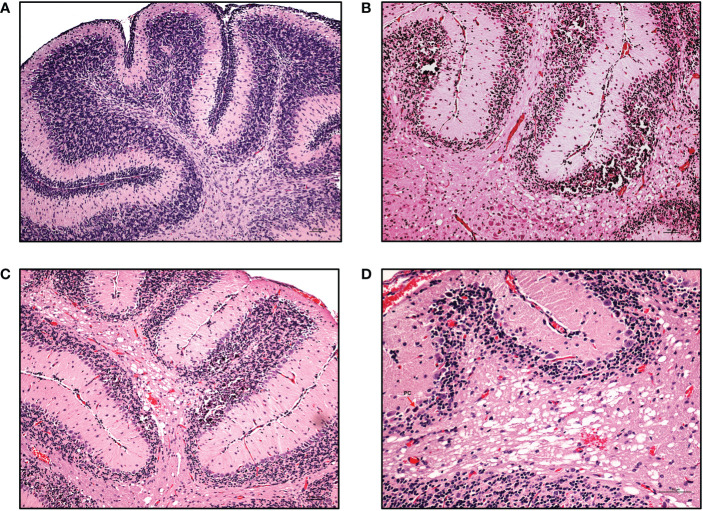
Cerebellar pathologies in ZIKV-infected neonatal hu-mice. Hematoxylin-eosin staining of the hippocampus of age-matched uninfected control and ZIKV-infected neonatal hu-mice. **(A)** The age-matched uninfected neonatal hu-mouse control shows a well-populated external (EGL) and internal (IGL) granular layer at 21 days post-engraftment. **(B)** The absence of an EGL and the under population of the IGL leading to the diminished overall cerebellar size with multifocal mineralization is seen in ZIKV-infected neonatal hu-mice. **(C)** The cerebellum shows the focal loss of the IGL in ZIKV-infected neonatal mice. **(D)** Magnified view of the cerebellar white matter vacuolation, status spongiosus and microscopic hemorrhages. Images **(A)**, **(B)** and **(C)** were taken at a magnification of 100x, while the magnification of image **(D)** was 200x. The black scale bar in each image represents 100 µm. Representative images are shown.

Clinically, CZS has been shown to encompass microcephaly with neural pathologies and ocular anomalies (57). Brain structure disruptions have been observed, particularly abnormal gyral patterns, thin cerebral cortices, larger amount of fluid spaces (porencephaly), calcification of subcortical regions, corpus collosum abnormalities, asymmetric and increased ventricles (ventriculomegaly and hydrocephalus), lesser amounts of white matter, and cerebellar hypoplasia (57–60). To determine if our model recapitulates brain anomalies similar to those seen clinically with CZS, we next characterized the neuropathology of the brain in these ZIKV-infected hu-mice. Many developmental abnormalities and virus-induced defects were noted. Histopathological findings of the hippocampus, cortex, striatum, ventricles, cerebellum, thalamus, brain stem, eyes, and spinal cord are summarized in **Table 1**. Normally, the pyramidal cell neurons of the hippocampus in an uninfected mouse are large, open-faced nuclei within the hippocampal formation (HCF) molecular layer (**Figures 5A, C**). However, in 10 out of 11 ZIKV-infected neonatal hu-mice, diffuse neuronal necrosis of the pyramidal neurons and subsequent collapse of the *cornu Ammonis* CA1, CA2, and CA3 layers of the HCF was observed (**Figures 5B, D**). In addition to bilateral necrosis of the hippocampus, we also observed focal hemorrhage and mineralization of the hippocampus in these ZIKV-infected neonatal hu-mice (**Figure 6A**). Similar pathologies were also observed in the dentate gyrus (data not shown). Histologically, the multifocal calcification was widespread as seen in several regions of the brain including the cerebral cortex, thalamus, striatum, and HCF (**Figure 6B**). This widespread neuronal necrosis and mineralization lead to focal disruption of the cytoarchitecture of the striatum basal nuclei (**Figure 6C**) and advanced mineral encrustation of neurons in the midbrain substantia nigra (**Figure 6D**). To some degree in all ZIKV-infected neonatal hu-mice, the cerebellum displayed extensive underpopulation to complete absence of the external granular cell layer (EGL) with focal loss of the internal granular cell layer (IGL) (**Figures 7B, C**) when compared to the age-matched uninfected control hu-mice (**Figure 7A**). This, in turn, led to diminished overall cerebellar size with multifocal mineralization (**Figures 7B, C**). On close observation, the cerebellar white matter showed extensive vacuolation, status spongiosus, and microscopic hemorrhages (**Figure 7D**). Histopathological findings were seen in the brain stem to varying degrees in 6 out of 11 hu-mice (**Table 1**). Retinal atrophy or retinal dysplasia was recorded in the eyes of 4 out of 11 hu-mice. However, there was little evidence of ZIKV pathology in the spinal cord.

**Table 1:** Histopathological findings in ZIKV-infected neonatal hu-mice.

Mouse #	Days Post-Engraftment	Hippocampus	Cortex	Striatum	Ventricles	Cerebellum	Thalamus	Brain Stem	Eyes	Spinal Cord
1	13	+CA3	++MF	+/-	–	+	+	–	–	–
2	13	++CA2, 3	++MF	+	+	++	+	+	–	–
3	14	++CA3	++D	++	–	++	+	+	+RA	–
4	14	+CA1, 3	++	++	+	++	+	–	++RD, HP	–
5	14	+/-	+	+/-	–	+	+/-	–	–	–
6	14	–	+	–	–	+/-	–	–	–	–
7	15	+CA3	++	+	+	+	+	–	–	–
8	17	++CA2, 3	+++	++	++	++	++	+	+RA	–
9	19	++CA1, 2, 3	++	+	+	++hypo	++	+	–	–
10	21	+++	+++	+++	+	++hypo	+	+	+	–
11	21	++	+	+	+/-	+	+	+/-	–	–

-, Negative (no histologic lesions/immunoreactivity detected in the sections examined); +/-, Equivocal or minimal immunoreactivity staining in less than 10% of the area of interest; +, Mild immunoreactivity staining in 10-25% of the area of interest; ++, Moderate immunoreactivity staining in 25-50% of the area of interest; +++, Marked immunoreactivity staining in >50% of the area of interest; MF, Multifocal; CA1, 2, 3, cornu Ammonis 1, 2, and 3 subfields of the hippocampus proper; D, diffuse; Hypo, Cerebellar Hypoplasia; RA, Retinal atrophy; RD, Retinal dysplasia; HP, Hypoplasia.

The # is used to identify and differentiate individual mice with their listed pathologies in the table.

**Figure 8:**
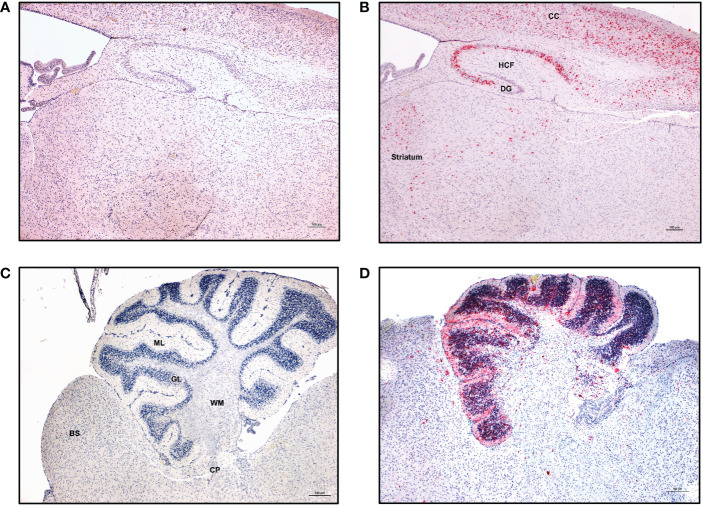
ZIKV-specific immunoreactivity in different regions of the CNS. Immunohistochemistry was performed on the paraffin embedded hemi-brain of ZIKV-infected neonatal hu-mouse 19 days post-engraftment using Flavivirus envelope protein (4G2) antibody or control negative antibody. Fast red or DAB were used as chromogens, and sections were counterstained with hematoxylin. **(A)** Control negative antibody stained sections. **(B)** Sections stained with 4G2 antibody showing strong immunoreactivity in the cerebral cortex (CC), hippocampus formation (HCF), dentate gyrus (DG), thalamus and striatum. **(C)** Cerebellum of an age-matched uninfected hu-mouse showing no immunoreactivity throughout all cerebellar layers. **(D)** Cerebellum from a ZIKV-infected neonatal hu-mouse at 19 days post-engraftment showing immunoreactivity in the molecular layer (ML), granular layer (GL) and the cerebellar peduncle (CP). Images were taken at 100X magnification with the black scale bar in each image representing 100 µm. Representative images are shown.

We next proceeded to evaluate the presence of viral antigens by immunohistochemistry using antibody reactive to the viral envelope in the cerebral cortex, hippocampal formation, dentate gyrus, thalamus, and striatum of ZIKV-infected neonatal hu-mice. Strong ZIKV antigen immunoreactivity was observed in the cerebral cortex, hippocampal formation, dentate gyrus, thalamus, and striatum (**Figures 8A, B**) in infected mice in contrast to controls. In particular, the molecular layer, granular layer, and cerebellar peduncle of the cerebellum all showed positive ZIKV antigen staining relative to controls (**Figure 8C, D**). Taken together, the above histopathological findings further confirmed that microcephaly and CZS could be effectively recapitulated in the ZIKV-infected neonatal hu-mouse model.

## 4 Discussion

Animal models are essential for testing novel vaccines or antiviral therapeutics against ZIKV. While many previous studies employing mouse, nonhuman primate, pig, sheep, hamster, rat, and chicken embryo models helped our understanding of ZIKV pathogenesis and immunity (23, 61, 62), deficiencies unique to each of these models left knowledge gaps on the potential viral adverse effects on the developing human immune system as well as on peri-and postnatal nervous system maturation (23, 61, 62). Also, the outcomes of prenatal infection showed considerable variation across animal models, though the causal relationship between ZIKV and CZS has been well studied (23).

Our current study addressed these important developmental questions by utilizing a novel neonatal humanized mouse model engineered with ZIKV-infected human HSC. Newborn mice were engrafted with infected CD34^+^ cells at 1-3 days post birth, which is comparable to 23-32 weeks of human gestation (second and third trimester of pregnancy) (63). Mechanistically, post-natal infection also permits evaluation of arrested/impaired neurological development seen in the infected newborn infant. This is particularly important given that humans are unique in having extended periods of neurological development throughout their childhood (63).

Striking gross pathologies of ZIKV in the infected neonatal humanized mice were seen compared to controls that included severe growth retardation and weight loss. At the time of euthanasia (13-21 days post-infection), these mice were found to have high levels of ZIKV viremia (>10^6^ RNA copies/mL; **Figure 1B**). Arrested development of various organs including the brain was evident. Clear neurological symptoms associated with microcephaly and CZS were also seen and are consistent with previous literature in other models (reviewed in (23)). Potential endocrinopathies resulting in multi-hormone deficiencies might have contributed to retarded growth of vital organs namely liver, lungs, bone marrow, spleen, and eyes (64). We also found that several pups had drastic ocular developmental defects, including retinal atrophy, retinal dysplasia, and hypoplasia. Mouse retinal development occurs within the first three weeks following birth resembling human retinal development during the third trimester of pregnancy (65). Our findings mimic the ZIKV-induced retinopathy observed by Li et al. in C57BL/6J mice inoculated with ZIKV at postnatal day 0 wherein ZIKV infection over time resulted in decreased retinal vascular coverage and density and induced progressive retinal degeneration and inflammation similar to that seen in infants with CZS (66).

Previously, we demonstrated that ZIKV infected BLT (bone marrow, liver, thymus mice) hu-mice inoculated with ZIKV (PRVABC59) displayed viremia that persisted for as long as 3-6 months in some mice similar to what has been observed in a subset of nonhuman primate and human cases (23, 45, 67–72). Robust viral reacting and neutralizing human antibodies were detected by a flow cytometry-based neutralization and radio-immunoprecipitation assays (45, 72). More importantly, human myeloid, B cells, and HSC in the bone marrow were found to be ZIKV infected posing interesting questions on broader Zika viral tropism with implications for viral pathogenesis and reservoir persistence. Thus far, apart from our previous findings (45, 72), experimental data is lacking on *in vivo* effects of ZIKV on various cells of the hematopoietic compartment. While ZIKV is shown to infect monocytes, macrophages, and dendritic cells (54–56, 73), potential viral effects on HSC lineage specific differentiation into end stage cells is largely unknown. Here, we showed that CD19^+^ B cells of the spleen, bone marrow, and peripheral blood as well as CD20^+^ cells in spleen and blood were significantly reduced.

Flow cytometry analysis showed that B cells in the spleen and bone marrow were infected with ZIKV, suggesting B cell levels may be reduced due to either apoptosis or arrested maturation or both. While ZIKV induced apoptosis was evaluated *in vitro* in various contexts (74), data has been lacking on the effect of the virus on fetal immune cell development *in vivo*. Lymphopoiesis begins at 6 weeks post-conception in the fetal liver (75). B cells appear in the fetal liver by 7 weeks during the first trimester, with a shift to the fetal bone marrow as the main site of B-lymphopoiesis during the second trimester (76, 77). Our *in vivo* data showed clear B cell reduction during ZIKV infection. Translating these findings to the human context *in utero*, adverse effects on B cells and their differentiation from the progenitor cells will have serious implications for viral pathogenesis and transfer of the virus to the CNS of the developing fetus. For example, failure to generate neutralizing antibodies due to B cell deficiencies will permit uncontrolled viral multiplication and spread leading to an increase in ZIKV crossing of the blood-brain barrier. Viral pathologies on the CNS will then result in the neuronal defects seen.

In addition to B cells, there was also a significant reduction in the number of CD34^+^ HSC found in the bone marrow, suggesting additional hematopoietic cell disruption. Interestingly, the CD14^+^ myeloid cells of the bone marrow, spleen and peripheral blood developed normally similar to age-matched controls, even though they were ZIKV positive in the spleen but not bone marrow. These migrating myeloid cells may be contributing to ZIKV spread in the fetus and subsequent CZS pathologies.

Besides our humanized mouse studies, a study by Roth et al., assessed ZIKV tropism in hematopoietic cells *in vitro* using adult CD34^+^ HSC from G-CSF mobilized blood as well as BM (78). When assessing the replication kinetics of French Polynesian ZIKV patient isolate (PF12/251013-18) in adult CD34^+^ HSCs, the authors detected ZIKV for up to two days in the supernatant but were unable to further detect ZIKV by either RNA or flow cytometry (data not shown) to detect the viral NS4B and envelope proteins during the subsequent days (78). In contrast, our *in vivo* studies found ZIKV infected CD34^+^ HSC in the bone marrow of humanized neonatal ZIKV-infected mice by flow cytometry. Additionally, we previously showed the presence of ZIKV infected CD34^+^ HSC in the bone marrow of adult BLT mice (45). We also determined the replication kinetics of ZIKV in CD34^+^ HSC *in vitro*, using the Puerto Rico strain (PRVABC59). Productive infection as assessed by RT-PCR for viral RNA in the culture supernatants persisted for 16 days (Data not shown). The different findings in these studies could be attributed to the differences observed *in vivo* of the ZIKV strains from the Asian lineage (23) as well as the inherent differences between adult and fetal HSC (79) that could affect viral interactions and replication kinetics. However, infection of fetal derived CD34^+^ HSC has more relevance to congenital and *in utero* ZIKV infection and the associated hematopathologies.

Zika virus infection disrupts the developing brain in infected human and animal fetuses due to the substantial damage it causes (80, 81). The plethora of congenital pathologic lesions usually precipitate devastating neuropathology including microcephaly, cognitive development due to cerebral hypoplasia, and blindness (81–83). In our study, we were able to mimic microcephaly syndrome with a smaller head circumference, overall brain hypoplasia and consequent neuronal syndromes similar to that seen in humans (9). Our model is unique in that we were able to document the neurological effects of ZIKV postnatally to determine how the virus elicits CZS. As mentioned previously, this is critical given that human newborns continue to develop neurologically throughout their childhood (63). As our mice were reconstituted on postnatal days 1-3 with infected CD34^+^ HSCs, this is comparable to ZIKV exposure during 23-32 weeks human gestational period. During this period, developmental changes intensify including pronounced mitotic activity of oligodendrocyte precursors (84–87), immune system ontogeny (88), and institution of the blood brain barrier (89, 90). The peak of brain development and maturation of the immune system occurs in the mouse at P7-P10, which compares to 36-40 weeks (third trimester) of gestation in humans (85, 91). Of particular importance in the current model is the arrested hippocampal formation and death of neuronal progenitor cells. Pyramidal neurons and dentate gyrus are among the earliest cortical neurons that develop, and their dysfunction will lead to cerebral hypoplasia (91, 92). Viral exposure of the pituitary gland of neonatal humanized mice has the potential to result in multiple endocrinopathies with thyroid and adrenal dysfunctions like those reported in Brazilian children during the 2015 epidemic (64). Intracranial calcification is among the major lesions that lead to behavior abnormalities in infant macaques and children (93, 94). Attesting to these viral-mediated nature of these conditions, viral antigens were detected in the areas of intracranial calcifications (ICC) most notably in the thalamus and the fasiculus retroflexus as seen in our model (95). The current study confirmed the neurotropism of ZIKV in humanized mice leading to a cascade of developmental defects in the head, particularly the brain. Semiquantitative histopathologic and immunohistochemical analyses of the full-term brain of the humanized mice can easily translate into human neural development in infants with CZS. Gross and histologic neuroanatomical findings revealed the presence of and adverse effect of ZIKV on the frontal lobes, midbrain, cerebellum and visual pathway.

In summary, using this unique neonatal humanized mouse model we evaluated the drastic effects of ZIKV on the developing nervous system and also established the adverse effects of the virus on human hematopoiesis, especially on terminal B cell differentiation. Based on these results, this model would also be useful to further dissect hematopoiesis by more closely examining questions like the impact ZIKV infection has on other cellular compartments and seeding reservoirs like the reproductive system postnatally. Thus, this neonatal humanized mouse model provides a novel platform to answer questions relating to ZIKVs impact on fetal development and immunity as well as testing novel drugs and the effect of passive immunity in conferring protection.

## Data availability statement

The original contributions presented in the study are included in the article/supplementary material. Further inquiries can be directed to the corresponding author.

## Ethics statement

The animal study was reviewed and approved by Colorado State University IACUC.

## Author contributions

KS, JC and LR-M conducted the experiments. KS, JC, RA and TA analyzed and interpreted the data. TA is responsible for analytical pathology and interpretation. KS, RA, TA and JC wrote the paper. RA is responsible for the overall conception of the project and for obtaining funding. All authors contributed to the article and approved the submitted version.
